# Quality of life among patients with cardiac disease: the impact of comorbid depression

**DOI:** 10.1186/s12955-020-01433-w

**Published:** 2020-06-17

**Authors:** Mandreker Bahall, George Legall, Katija Khan

**Affiliations:** School of Medicine, Faculty of Medical Sciences, University of the West Indies, Eric Williams Medical Sciences Complex, Mt Hope, House #57 LP 62, Calcutta Road Number 3, Mc Bean, Couva, Trinidad Trinidad and Tobago

**Keywords:** Depression, Quality of life, Cardiac comorbidities, PHQ-9, SF12, PCS, MCS

## Abstract

**Background:**

Patients with cardiac disease with or without depression may also have major physical and mental problems. This study assesses and compares the quality of life (QOL) of patients with cardiac disease with and without depression and accompanying comorbidities.

**Methods:**

A cross-sectional study was conducted with a convenience sample of 388 patients with cardiac disease. The 12-item Short-Form (SF-12)-patient was used to measure physical component scale (PCS) and mental component scale (MCS) QOL, and the Patient Health Questionnaire (PHQ-9) was used to measure depression. The Charlson Comorbidity Index was used to estimate 10-year survival probability. Descriptive statistics, analysis of covariance (ANCOVA), chi-square tests, and binary logistic regression were used for analysis.

**Results:**

The prevalence of minimal to mild depression was 65.7% [(95% CI (60.8, 70.4)] and that of moderate to severe depression was 34.3% [95% CI (29.6, 39.2)]. There was no significant association between the level of PHQ-categorised depression and age (*p* = 0.171), sex (*p* = 0.079), or ethnicity (*p* = 0.407). The overall mean PCS and MCS QOL was 32.5 [95% CI (24.4, 40.64)] and 45.4 [95% CI (44.4, 46.4)], respectively, with no significant correlation between PCS and MCS [r (Pearson’s) = 0.011; *p* = 0.830)]. There were QOL differences among the five PHQ categories (PCS: *p* = 0.028; MCS: *p* ≤ 0.001) with both MCS and PCS decreasing with increasing depression. ANCOVA (with number of comorbidities as the covariate) showed a significant age × ethnicity interaction for PCS (*p* = 0.044) and MCS (*p* = 0.039), respectively. Young Indo-Trinidadians had significantly lower PCS than did Afro-Trinidadians, while the converse was true for MCS. Depression, age, and number of comorbidities were predictors of PCS, while depression, age, and sex were predictors of MCS.

**Conclusions:**

Increasing severity of depression worsened both PCS and MCS QOL. Age and level of clinical depression predicted QOL, with number of comorbidities predicting only PCS and sex predicting only MCS. Efforts must be made to treat depression in all age groups of patients with cardiac disease.

## Background

Quality of life (QOL) is a major outcome indicator of patients with cardiac disease with or without comorbidities such as depression, other psychosocial factors, and a multitude of chronic non-communicable diseases. Depression, defined by the World Health Organization (WHO) as “a common mental disorder, characterised by sadness, loss of interest or pleasure, feelings of guilt or low self-worth, disturbed sleep or appetite, feelings of tiredness, and poor concentration” [1], is quite prevalent worldwide. It can be “long lasting or recurrent, substantially impairing a person’s ability to function at work or school, or cope with daily life” [1]. Depression remains one of the most prevalent diseases globally, with the 12-month and lifetime prevalence of major depressive disorder being 10.4 and 20.6%, respectively, in the US [2]. In Trinidad and Tobago, depression accounted for 30% of the reported cases of mental illnesses [3]. Among patients with cardiac disease, depression ranges between 20 and 40% [4]. In Trinidad, 40% of stable (i.e. without deterioration, and able to perform basic activities of daily living) patients with cardiac disease were found to have depression [5].

Depression is associated with worsened QOL (physical, mental, and social), [6–8] medical morbidity, and mortality [9]. A strong association has been observed between symptoms of depression and health status, symptom burden, physical limitation, QOL, and overall health among patients with coronary artery disease (CAD) [10]. Depression influences lifestyle habits such as smoking, eating, exercising, getting along with family members, social life, and work [4, 11]. It can lead to lowered productivity, personal losses, and increased family and state burden. Other accompanying cardiovascular risks can further compromise patient QOL [12–15]. Many studies in developing countries have reported on poor QOL experienced by patients with cardiac disease with depression [16] who are two times more likely to die after a cardiac event [17]. Studies on QOL among patients with cardiac disease with depression have not been reported in Trinidad and Tobago. This study sought to examine and compare QOL among, and between, patients with cardiac disease with or without depression admitted to public tertiary health institutions in Trinidad and Tobago.

## Methods

This was a cross-sectional study, the target population being all patients presenting with cardiac disease at the three teaching hospitals in Trinidad and Tobago during the period November 2015 to March 2016. The population from which the sample was taken comprised all patients attending the cardiac clinic from the largest teaching hospital because by the time of study initiation, approval had been granted only by the largest hospital where the study was conducted. The hospital is a 745-bed facility that offers free public healthcare to a catchment of 600,000 persons. Annually, there are 46,785 admissions to this hospital, of which 15,339 (32.8%) are medical admissions (2010) [18]. Because of the protocols used for patient admission in these three hospitals, there is no reason to expect that the patients treated for CVD at this institution significantly differed from those treated in the non-participating hospitals during the study period in any characteristic (age, sex, and ethnicity) that would affect the findings of the study due to non-participation.

To be eligible for participation in the study, patients were to have been diagnosed with cardiac disease at least 3 months prior to the start of the study. The patients’ medical records in the cardiac clinic were used to create a sampling frame from which patients were selected. Inclusion criteria were 18 years of age or older and willingness to communicate for approximately 20 min. Exclusion criteria were confusion (incoherence), refusal to participate, poor health status (signs of mental and physical exhaustion), and unavailability at the time of selection. A sample size of 396 patients was computed to be the minimum needed in order to estimate the prevalence of depression within the target population with a 5% margin of error using simple random (probability) sampling [19].

**Fig. 1:**
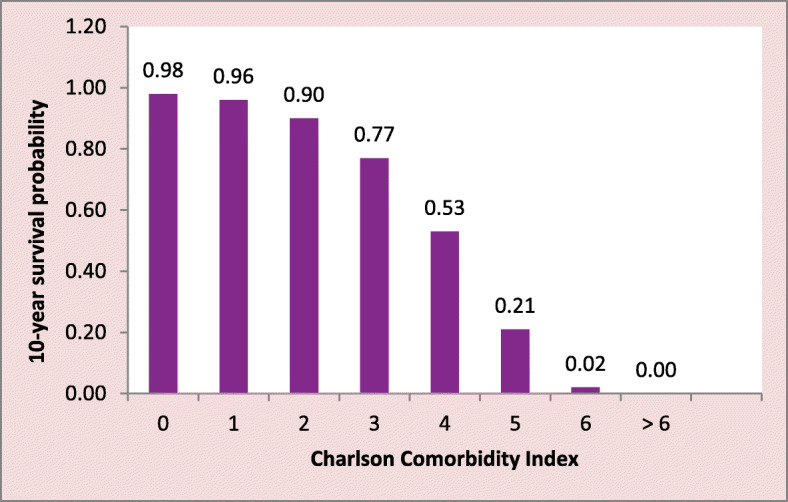
Charlson Comorbidity Index and the corresponding 10-year survival probability

Two data collection instruments, namely, the 12-item Short-Form 12 patient questionnaire to measure QOL and the Patient Health Questionnaire (PHQ-9), were used to measure depression. In addition, selected demographic variables such as age, sex, ethnicity, and education were included in the questionnaire. In keeping with standard depression research guidelines, patients with PHQ-9 scores ≥10 (out of 27) were classified as having clinically significant depression [20]. SF-12 data were used to measure both Physical QOL, i.e. the physical component score (PCS) and Mental QOL, i.e. the mental component score (MCS). Lower mean scores indicate poorer QOL. The Charlson Comorbidity Index [21] was used to classify patients according to their estimated 10-year survival probability (See Fig. 1). The Charlson Comorbidity Index, which is the probability of 10-year survival, was preferred to the number of comorbidities, which provides a measure of the prevalence of the number of comorbidities. It is also a more appropriate covariate when comparing QOL means using analysis of covariance (ANCOVA).

The SF12 comprises 12 questions on the general rating of one’s health, limitations imposed by current health status on moderate activities and climbing several flights of stairs, experiencing problems with work or regular daily activities due to physical health and emotional problems, how pain interferes with one’s normal work, assessing patient emotional state during the past 4 weeks and how much of the time has physical health or emotional health interfered with social activities. The SF12 yields two summary measures, namely, a Physical Component (PCS QOL) and a Mental component (MCS QOL), which were generated using standardised scoring guidelines [22, 23].

SPSS, version 21 (IBM Corp., Armonk, NY), was used for data analysis, by means of both descriptive and inferential methods. Descriptive methods included determining frequency and percentage distributions and summary statistics (means and standard deviations). Inferential methods included 95% confidence intervals (CI), hypothesis testing, and prediction (regression analysis). ANCOVA was used to test the equality of mean PCS and mean MCS QOL scores, using the Charlson Comorbidity Index as a covariate. McNemar’s test of paired proportions was used to test the association between self-reported depression and SF12-diagnosed depression. Microsoft EXCEL was used to produce tables, graphs, and charts.

## Results

### Demographics

By the end of the data collection period, 388 (98.0%) of the 396 patients were surveyed. Patients were predominantly female (*n* = 207, 53.4%), aged 65–74 years (*n* = 121, 31.2%), and Indo–Trinidadian (*n* = 280; 72.2%; Table 1).

**Table 1:** Frequency and percent distribution of demographic variables

Variable	n	%
**Sex**		
Male	181	46.6
Female	207	53.4
Age group (years)		
< 35	11	2.8
35–44	24	6.2
45–54	50	12.9
55–64	97	25.0
65–74	121	31.2
**≥** 75	85	21.9
Ethnicity		
Afro-Trinidadian	83	21.4
Indo-Trinidadian	280	72.2
Other	25	6.4

**Fig. 2:**
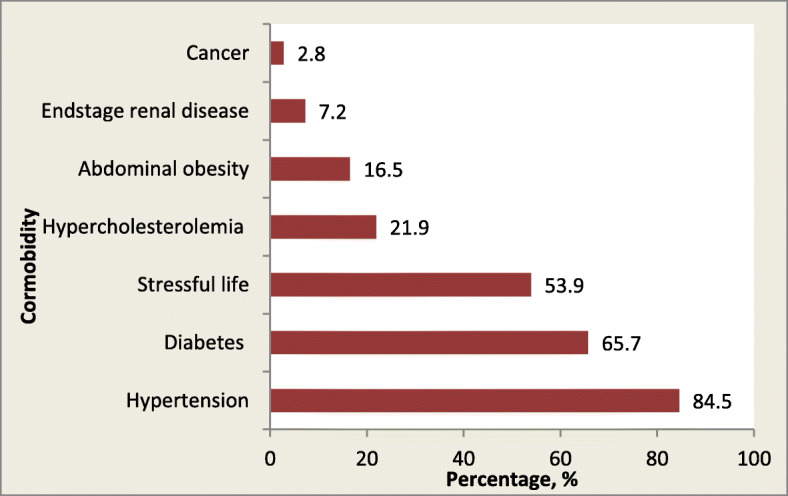
Prevalence of comorbidities among patients with cardiac disease

The overall mean age was 64.1 ± 13.37 years; male participant age was 62.4 ± 14.10 years, and female participant age was 65.6 ± 12.55 years, with women being significantly older than men (*p* = 0.019). The difference between the mean age of Afro–Trinidadian and Indo–Trinidadian patients was not significant (*p* = 0.877). Hypertension was the most prevalent comorbidity at 84.5% [95% CI (80.5, 88.0)], followed by diabetes at 65.7% [95% CI (60.8, 70.4)] and hypercholesterolaemia at 21.9% [95% CI (17.9, 26.4)]. Cancer was the least prevalent comorbidity at 2.8% [95% CI (1.4, 5.0)], and self-claimed stressful life was reported by 53.9% [95% CI (48.8, 58.9); Fig. 2]. In addition, 52.6% of participants had a family history of ischemic heart disease (IHD), 19.3% had a history of stroke or transient ischaemic attack, and 5.6% had peripheral vascular disease. Cardiac investigations performed included angiography (31.7%), coronary artery bypass grafting (7.7%), and angioplasty (7.5%).

**Fig. 3:**
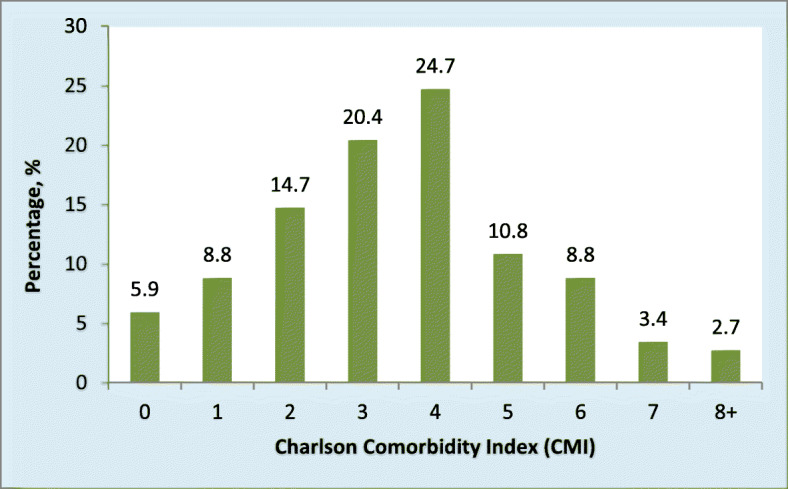
Charlson Comorbidity Index distribution of patients

The number of comorbidities ranged from 0 (*n* = 37, 9.5%) to 4 (*n* = 2, 0.5%) [mean: 1.6 ± 0.782, median: 2, mode: 2 (*n* = 207, 53.4%)]. Figure 3 shows the percentage distribution of the Charlson Comorbidity Index. As seen, 4-year CVD (*n* = 96; 24.7%), corresponding to a 10-year CVD survival probability of 53%, was the index with the highest prevalence. Figures 1 and 3 together show that 5.9% [95% CI (3.8, 8.8)] of the patients had a 98.0% 10-year survival probability (Charlson Comorbidity Index = 0) and 6.1% [95% CI (2.6, 6.9)] had a zero 10-year survival probability.

Only 7.5% [95% CI (5.1, 10.6)] of the patients lived alone; 13.1% [95% CI (9.9, 16.9)] lived with a spouse only, while 20.2% [95% CI (16.2, 24.4)] lived with a relative, and 49.2% [95% CI (44.1, 54.3)] lived with a spouse and at least one relative.

### Physical and mental quality of life

**Fig. 4:**
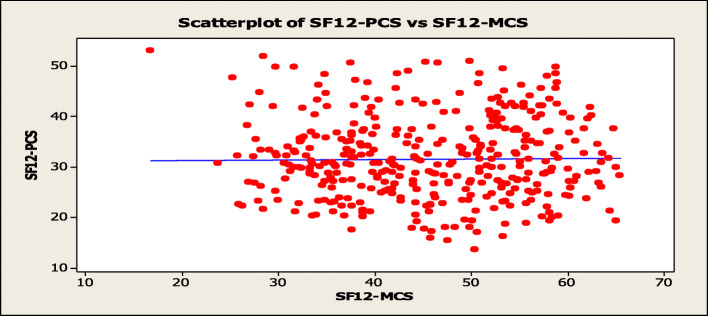
Scatterplot of PCS vs. MCS

The PCS and MCS from the SF12, demographics, and Charlson Comorbidity Index are shown in Table 2. The overall mean PCS and MCS QOL were 32.5 [95% CI (24.4, 40.64)] and 45.4 [95% CI (44.4, 46.4)], respectively. The scatterplot of PCS vs. MCS with the linear regression line (Fig. 4) showed no discernible linear or non-linear pattern or trend. Bivariate correlation analysis showed a Pearson coefficient (r) of 0.011 (*p* = 0.7830) for MCS and PCS.

**Table 2:** SF-12 domain scores by patient characteristics

Variable	QOL: Mean (SD)	
	PCS	MCS
Sex		
Male	32.7 (8.60)	47.6 (10.07)
Female	30.6 (7.64)	43.5 (10.13)
Age group (years)		
< 35	37.2 (9.86)	43.5 (15.01)
35–44	35.2 (8.53)	44.6 (11.43)
45–54	34.0 (7.36)	44.8 (9.84)
55–64	32.0 (8.84)	45.4 (10.74)
65–74	29.9 (7.72)	45.7 (9.61)
**≥** 75	30.3 (7.18)	45.9 (9.61)
Ethnicity		
Afro	33.7 (8.17)	47.9 (9.89)
Indo	30.7 (7.78)	44.7 (10.34)
Other	33.6 (10.62)	45.1 (10.21)
Charlson Comorbidity Index		
< 3	35.4 (8.18)	46.4 (11.34)
3–4	30.8 (8.27)	44.7 (9.85)
5–6	28.8 (6.24)	45.9 (9.63)
7+	26.4 (3.53)	44.7 (10.70)

## Depression: self-reported and PHQ diagnosed

The overall prevalence of PHQ-categorised depression was 78.4% [(95% CI (73.9, 82.3)] ranging from mild [38.4%; 95% CI (33.5, 43.4)] to severe [2.3%; 95% CI (1.1, 4.4)]. At the same time, the prevalence of clinical depression (PHQ ≥ 10) was 34.3% [95% CI (29.6, 39.2)] compared to a 38.4% [95% CI (33.5, 43.8)] prevalence of self-reported depression (Table 3). The difference was not significant (McNemar’s test of equality of paired proportions; *p* = 0.171). Additionally, chi-square analysis showed no association between clinical depression level and age (*p* = 0.171), sex (*p* = 0.079), or ethnicity (*p* = 0.407).

**Table 3:** Self-reported and clinical depression (numbers and percents of patients)

Self-reported depression: n (%)	Clinical Depression: n (%)		
	No	Yes	Total
No	187 (48.2)	52 (13.4)	61.6
Yes	68 (17.5)	81 (20.9)	38.4
Total	65.7	34.3	

### Depression/comorbidities and quality of life

ANCOVA with number of comorbidities as the only covariate showed significant differences in the mean MCS between patients in the five PHQ depression categories (*p* = 0.029) and a significant ***Age*** × ***Ethnicity*** interaction (*p* = 0.045). The adjusted means and corresponding standard deviations, along with the number of patients, are shown in the second column of Table 4. As shown, patients with no depression had higher mean PCS than those in every depression category and the mean MCS decreased with increasing levels of depression. This would be later confirmed by regression analysis.

**Table 4:** Adjusted PCS and MCS means by PHQ categories

		QOL Component: Mean (SD)	
PHQ Category		PCS	MCS
None	84	36.3 (8.11)	53.7 (6.50)
Mild	149	31.3 (7.42)	47.9 (9.45)
Moderate	106	29.5 (8.03)	40.3 (7.84)
Moderately Severe	40	27.8 (6.79)	35.7 (7.31)
Severe	9	31.1 (9.23)	31.8 (10.62)

**Fig. 5:**
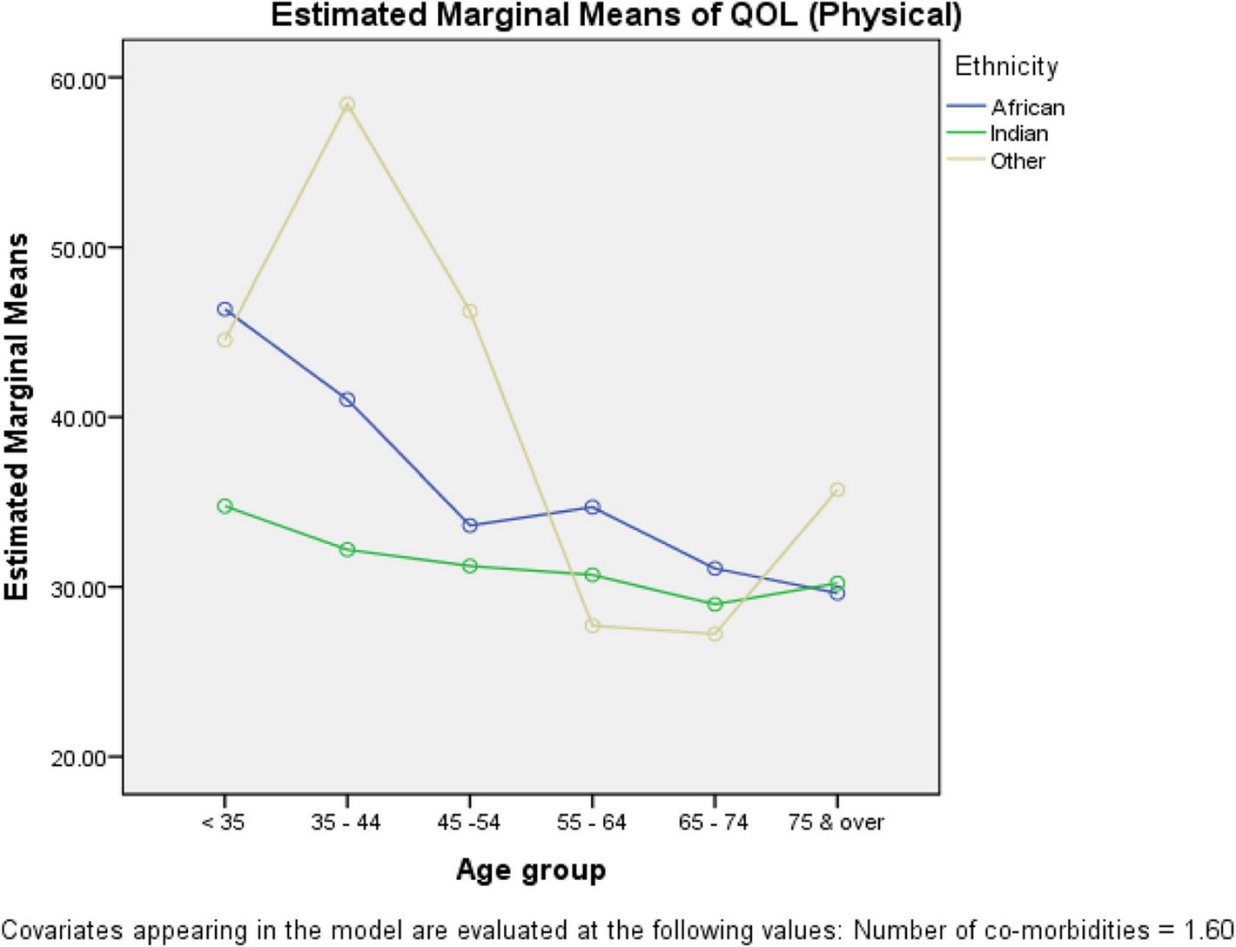
*Age* × *Ethnicity* interaction plot (PCS)

Fig. 5 shows the ***Age*** × ***Ethnicity*** interaction plot of the mean PCS. As shown, for patients under 35 years of age, patients aged 35–44 years, and patients 55–64 years of age, the mean PCS of patients of Afro–Trinidadian descent was higher than that of those of Indo–Trinidadian descent and that the difference in mean PCS between these two ethnic groups was not significant for patients aged 45–54, 65–74, or 75 years or older.

**Fig. 6:**
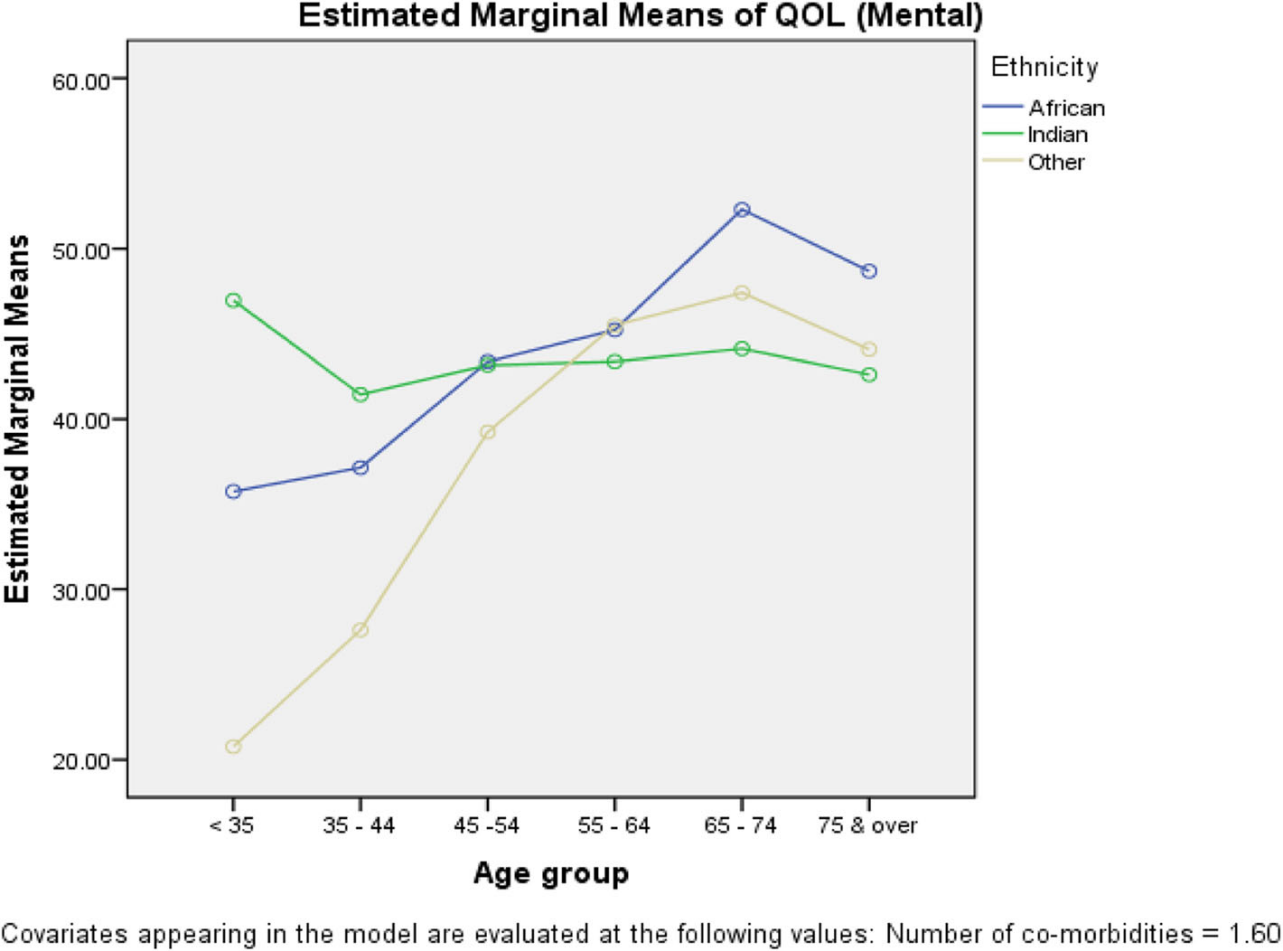
*Age* × *Ethnicity* interaction plot (MCS)

Similar ANCOVA methods were used to test the equality of mean MCS, specifically for the PHQ depression categories (*p* ≤ 0.001) and the significant ***Age*** × ***Ethnicity*** interaction (*p* = 0.027). Adjusted means and corresponding standard deviations along with the number of patients are shown in the second column of Table 6; the ***Age*** × ***Ethnicity*** interaction plot is shown in Fig. 6.

The last column of Table 4 also shows that patients with no depression had higher mean MCS than those in every depression category and that mean MCS decreased with increasing level of depression; this was confirmed using regression methods.

Figure 6 shows the ***Age*** × ***Ethnicity*** interaction plot of the mean MCS. As shown, for patients under 35 years of age and patients aged 35–44 years, the mean MCS was higher among those of Indo–Trinidadian than among those of Afro–Trinidadian descent. There were no significant differences among the two groups for patients aged 45–54 and 55–74 years. However, for patients 65–74 and patients 75 years or older, the mean MCS of patients of Afro–Trinidadian descent was significantly higher than that of their Indo–Trinidad counterparts.

### Predictors of quality of life

Multivariable regression analysis was used to fit the following equation to the PCS and MCS data:

$$ y={\beta}_0+{\beta}_1{x}_1+{\beta}_2{x}_2+{\beta}_3{x}_3+{\beta}_4{x}_4+{\beta}_5{x}_5+{\varepsilon}_i,\mathrm{where} $$

$$ {x}_1=\kern0.5em \mathrm{Age},\kern0.5em {x}_2=\mathrm{Sex},\kern0.5em {x}_3=\mathrm{Ethnicity},{x}_4=\kern0.5em \mathrm{Number}\ \mathrm{of}\ \mathrm{comorbidities}, $$

$$ {x}_5=\mathrm{PHQ}\ \mathrm{categories} $$

The regression coefficients for each model are shown in Table 5 for PCS and MCS, respectively.

**Table 5:** Regression coefficients (dependent variables PCS and MCS)

QOL Component	Variable	Coefficient (*β*_*i*_)	$se\left({\overset{\wedge }{\beta}}_i\right)$	*p*-value
PCS	Constant	44.96	2.016	≤ 0.001
	Age	−0.114	0.030	≤ 0.001
	Sex	− 0.659	0.783	0.401
	Ethnicity	−0.340	0.559	0.543
	Comorbidities	−1.643	0.527	0.002
	PHQ category	−2.09	0.390	≤ 0.001
MCS	Constant	51.73	2.193	≤ 0.001
	Age	0.070	0.033	0.033
	Sex	−2.624	0.852	0.002
	Ethnicity	−0.782	0.608	0.199
	Comorbidities	−0.548	0.574	0.340
	PHQ category	−5.856	0.424	≤ 0.001

As seen in Table 5, of the five independent variables only age (*p* ≤ 0.001), number of comorbidities (*p* = 0.002), and PHQ category (*p* ≤ 0.001) were predictors of the PCS. For the MCS, the predictors were age (*p* = 0.033), sex (*p* = 0.002), and PHQ category (*p* ≤ 0.001). Coefficients of the two reduced models, with corresponding standard errors, are given in Table 6.

**Table 6:** Regression coefficients (dependent variables PCS and MCS)

QOL Component	Variable	*β*_*i*_	$se\left({\overset{\wedge }{\beta}}_i\right)$	*p*-value
PCS	Constant	46.67	2.067	≤ 0.001
	Age	−0.116	0.030	≤ 0.001
	Comorbidities	−1.709	0.521	0.001
	PHQ category	−2.134	0.387	≤ 0.001
MCS	Constant	50.86	2.110	≤ 0.001
	Age	0.070	0.031	0.048
	Sex	−2.749	0.843	0.001
	PHQ category	−5.954	0.417	≤ 0.001

Therefore, the respective reduced prediction equations are:

$$ {\overset{\wedge }{y}}_{PCS}=46.67-0.116{x}_1-1.709{x}_4-2.134{x}_5, $$

and

$$ {\overset{\wedge }{y}}_{MCS}=50.86-0.070{x}_1-2.749{x}_2-5.954{x}_5 $$

## Discussion

Overall, the mean PCS and MCS QOL scores in our study were 32.5 [95% CI (24.4, 40.64)] and 45.4 [95% CI (44.4, 46.4)], respectively. QOL scores worsened with increasing depression and were lower than those reported for a standardised US population. Lower QOL scores among patients with cardiac disease is a cause of national concern because of the high prevalence of depression among patients with cardiac disease in Trinidad [5]. Standard reference QOL values in Trinidad are not available. The relationship between comorbid depression and QOL is complicated, although, in general, the severity of depression is related to worsening QOL. Weiss et al. concluded that there was a strong association between depressive symptoms and reduced health related QOL [24]. Patients with depression after myocardial infarction had a higher probability of having a poor QOL [25]. This contradicts the findings of AbuRuz et al. [26] and may be related to the disease itself and its severity [12], the perception of the disease [27], or its consequences [28, 29] (social, psychological, and economic). Although physical health is affected by depression, the effect is less consistent and varies with the comorbid medical condition [30]. Physical health deterioration in patients with cardiac disease with depression was reported by Ruo et al. [10]. Conversely, Allabadi et al. reported weak correlations between the Cardiac Depression Scale and SF-12-MCS and PCS scores [31].

Numerous studies have reported on QOL among patients with other comorbidities. Our study revealed that PCS QOL was generally worse with increasing number of comorbidities. Such findings were seen in several studies [32–34]. Patients with CVD with osteoarthritis comorbidity were found to have poorer physical health [35] and those with chronic obstructive pulmonary disease exhibited poorer physical fitness [36]. Shad et al. also reported that the MCS was significantly lower in patients with multiple comorbidities, with the MCS being related to sex and educational level [32]. Accompanying comorbidities are of concern, as our study reveals there was a considerable number of comorbidities, ranging from 0 to 4, among patients with cardiac disease.

Studies have shown an association between the presence of comorbidities in patients with heart failure (HF) and a lower QOL, leading to increased mortality risk [37]. Comorbid diabetes and depression were associated with decreased survival rates and increased rehospitalisation in patients with HF [38].

Studies have revealed that having a higher comorbidity burden had a higher association with more depressive symptoms and with poorer physical functioning, physical limitations, and poorer general health [39, 40].

### Interaction with age, ethnicity, and sex

Our study revealed that there was a significant age and ethnicity interaction, with worsening QOL scores experienced with increasing depression. PCS QOL was higher among Afro–Trinidadian than among Indo–Trinidadian patients younger than 35, 35–44, and 55–64 years of age, but the difference was not significant among patients 75 years of age or older (Fig. 5). In contrast, with respect to MCS QOL, the reverse was generally true among Indo–Trinidadians, where patients younger than 35 years of age and patients aged 35–44 years showed higher MCS than did Afro–Trinidadian patients, and the converse was true among patients 75 years of age or older (Fig. 6). The lower physical composite scores among young Indo–Trinidadians may have resulted from a sedentary lifestyle and possible over protection from relatives. Beata et al. reported that age had no effect on QOL [8]. However, AbuRuz et al. found age to be an independent predictor of poor QOL citing that anxiety and depression scores and disease severity increased with age, which would have led to a reduction in physical and mental abilities, resulting in the lower QOL [41] found among patients with HF.

### Predictors and quality of life

Our study revealed that age, depression, and number of comorbidities were predictors of PCS and that age, depression, and sex were predictors of MCS. The better QOL experienced by men may be related to our male-dominant society or the greater exposure of men to support services in terms of social and physical activities. Najafi et al. also reported that male sex was an independent physical component predictor of higher QOL [42]. In contrast, Nesbitt et al. argued that male sex was associated with lower QOL [43] and that the sex difference was due to differences in family responsibility and gender roles and the responsibility of looking after one’s own health. Dickens et al. reported that depression was a predictor of physical health-related QOL in patients with coronary heart disease (CHD) [44]. Similarly, another study observed significantly poorer health-related QOL among patients with CHD who had anxiety and depression in all domains, particularly, in physical functioning and role functioning [45]. AbuRuz reported that predictors of high depression levels among patients with heart conditions were severity of disease, job status, and social support, which all led to poorer QOL [41]. Wang et al. reported that depression is a predictor of mental health-related QOL among patients with acute myocardial infarction [46]. Cruz et el found that depressed patients with IHD had lower QOL scores than did their non-depressed counterparts in the mental health, emotional, and social functioning domains [47]. Age and sex were predictors of MCS QOL. Hawkes et al. listed younger age as a predictor [48] and McBurney et al. found age below 65 years to be associated with low MCS-12 scores [49]. El-baz et al. [50] and Duenas et al. [51] both found female sex to be a strong predictor of and have an association with lower mental health, respectively.

Depression among patients with cardiac disease must be identified especially when the principal concern is accompanied by comorbidities. This necessitates a simple depression screening tool, referral systems, and treatment strategies for patients.

### Limitations

This was a single-centre study with a relatively small, convenience sample. Our results largely depended on self-reports and recall, which can be unreliable for many patients. Although patients were willing to share their feelings, many responses could have been exaggerated or under-reported. The sample was mainly of a lower socioeconomic background who seeks support from the free public health system. The stressors posed by personal economic conditions may also be exacerbating the prevalence of depression and as such, generalisation of the prevalence of depression in patients with CVD may prove difficult. Nonetheless, this study produced robust findings, highlighting the relationship between depression and QOL in patients with cardiac disease.

## Conclusions

Psychosocial factors such as depression and comorbidities worsen physical and mental health QOL. There were significantly age and ethnicity interaction effects, with young Indo–Trinidadians being more greatly affected than young Afro–Trinidadians with regard to PCS, while the converse was found for MCS. As other studies [14] have shown depression worsens QOL, every effort must be made to identify patients with depression and patients who have comorbidities such as diabetes mellitus, hypertension, and hypercholesterolaemia.

## Data Availability

The data that support the findings of this study are available from the corresponding author on request.

## Abbreviations

ANCOVA: Analysis of covariance
ANOVA: Analysis of variance
CAD: Coronary artery disease
CHD: Coronary heart disease
CI: Confidence interval
CVD: Cardiovascular disease
HF: Heart failure
IHD: Ischemic heart disease
MCS: Mental Component Scale
PCS: Physical Component Scale
PHQ: Patient Health Questionnaire
QOL: Quality of Life
SF12: 12-Item Short Form
